# A pre-trained language model approach for triaging surgical patients for preoperative anesthesia clinics

**DOI:** 10.1007/s10877-025-01401-z

**Published:** 2025-12-24

**Authors:** Nicole Y. Xu, Onkar Litake, Jeffrey L. Tully, Minhthy N. Meineke, Anika Sinha, Megan Meyer, Rodney A. Gabriel

**Affiliations:** 1 School of Medicine, University of California, San Diego, La Jolla, CA 92037, USA; 2 Division of Perioperative Informatics, Department of Anesthesiology, University of California, San Diego Campus Point Dr, La Jolla, CA 9400, USA; 3 Department of Biomedical Informatics, University of California, San Diego Health, La Jolla, CA 92037, USA

**Keywords:** Language models, Natural language processing, Artificial intelligence, Preoperative medicine, Preoperative care clinic

## Abstract

**Purpose:**

Preoperative anesthesia evaluation is a crucial step in ensuring patient safety and optimizing perioperative care. A heterogenous patient population requiring varying levels of assessment often leads to inefficiencies and additional resource allocation. This study proposes using pre-trained language models to assist in triaging the appropriate degree of preoperative anesthesia evaluation for surgical patients.

**Methods:**

Retrospective institutional data were obtained from surgical patients evaluated at a single center preoperative anesthesia care clinic. The performance of four pre-trained language models (RoBERTa, BERT, ClinicalBERT, and PubMedBERT) in the classification of which patients would be appropriate for a nursing preoperative phone call versus in-person clinician evaluation was assessed using F1-score, area under the receiver operating characteristics curve (AUC), specificity, sensitivity, and average precision. For each pre-trained language model, three different data input combinations were assessed: (1) diagnosis codes (D); (2) clinical text data (N); and (3) diagnosis codes and clinical text (D + N). The data were split into training (75%) and test set (25%).

**Results:**

There were 1,761 unique patients, with an average of 12 notes per patient and a total of 46,922 clinical documents, included in the analysis. The AUC range between the four language models was highest in the D + N analyses (0.70 – 0.74), lower in the N analyses (0.58 – 0.73) and lowest in the D analyses (0.57 – 0.62). RoBERTa had the highest score compared to the other language models for all data types.

**Conclusions:**

Automating integrated analysis using pre-trained language models to aid in preoperative triaging could enhance accuracy and efficiency at scale, reducing manual review and provider burden.

## 1 Introduction

Preoperative anesthesia evaluation of surgical patients is important to provide tailored care to optimize the medical condition of each patient. It is estimated that the occurrence of a postoperative major adverse cardiac event such as stroke, myocardial infarction, and cardiovascular death is around 20% in high-risk patients undergoing non-cardiac surgery [1]. Preoperative assessment and subsequent management aims to mitigate these adverse events. Preoperative care clinics have been established to catalogue co-morbid disease, stratify perioperative risks, and facilitate interventions or consultations prior to surgery [2]. Surgical patient volume is increasing dramatically with the expanding aging population and emerging surgical technologies [3]. This results in a similarly increased demand for preoperative evaluation that is difficult to meet due to the expansion of electronic health record (EHR) data management and limited healthcare resources, potentially leading to overburdened clinicians as well as less effective surgical optimization and worse outcomes [4, 5]. Inadequate preoperative evaluations may also lead to case cancellations on the intended day of surgery, significantly reducing patient access to care [6].

Resource allocation and optimization within preoperative anesthesia clinics is vital [7]. Clinics need to triage the degree of preoperative assessment needed for each patient (e.g. nursing preoperative phone call versus in-person or telehealth visit by an advanced practice provider or anesthesiologist), while balancing resource limitations such as staffing levels and clinic space availability [8]. The decision-making process to determine the most suitable level of anesthesia evaluation is nuanced and influenced by diverse factors such as the patient’s medical history, existing comorbidities, and the complexity of the planned surgical procedure.

In recent years, advances in natural language processing (NLP) and machine learning have sparked interest in language model approaches to enhance healthcare decision-making and reduce provider burden [9]. The objective of this study was to develop pre-trained language model-based classifiers that solely utilized structured data (e.g. diagnosis codes), solely utilized unstructured data (e.g. clinical notes), or utilized both structured and unstructured data to aid in triaging the degree of preoperative evaluation required for surgical patients. We hypothesized that the incorporation of both clinical notes and structured diagnosis codes into our classifier would improve model performance compared to clinical notes or diagnosis codes alone. This would demonstrate that automation of preoperative triaging would benefit from leveraging both types of data.

## 2 Methods

This retrospective study was approved by our institution’s review board, Human Research Protections Program, at the University of California, San Diego for the collection of data from the EHR system. As this was not human subjects research, the informed consent requirement was waived. Institutional data was obtained retrospectively from surgical patients evaluated at the institution’s preoperative anesthesia care clinic in March of 2023. The manuscript followed the Strengthening the Reporting of Observational Studies in Epidemiology guidelines.

### 2.1 Study population and outcomes assignment

At the institution’s preoperative anesthesia clinic, patients are triaged to either: (1) phone call evaluation by a nurse, or (2) in-person or telehealth evaluation by an anesthesiologist or advanced practice provider (e.g. nurse practitioner or physician assistant). A patient required an in-person or telehealth visit if they fulfilled any of the following criteria: (1) scheduled for a high-risk surgery, (2) body mass index greater than or equal to 50 kg/m^2^, (3) hospitalized within the last three months, (4) takes a high-risk medication, (5) has an implantable cardiac device, (6) is unable to perform greater than four metabolic equivalents, (7) has a known or potential difficult airway or other anesthesia-related problem, and (8) has severe end-organ disease (e.g. decompensated heart failure, severe coronary artery disease, major cardiac valve disease, severe pulmonary disease, end-stage renal disease, or end-stage liver disease). A committee of three anesthesiologists who regularly staff the institution’s preoperative clinic (authors JT, MM, and MM) designated each procedure current procedural terminology code as either low, medium, or high risk to determine the inclusion criteria of high-risk surgery. A high-risk medication list was created by the same committee and included medications such as anti-coagulants and insulin. If a patient did not meet any of these criteria, they would be assigned to receive a preoperative nursing phone call. The committee also reviewed all patient records in this study population and manually determined whether each patient would be appropriate for a nursing preoperative phone call evaluation. For each patient, all clinical notes and diagnosis codes (International Classification of Diseases, 9th and 10th edition) were queried from the EHR system (EPIC, Verona, WI). There were no missing data as we assumed that lack of presence of a diagnosis code meant the patient did not have the corresponding comorbidity. In clinical practice, the institution’s preoperative clinic nursing team triages patient’s evaluation visit type.

### 2.2 Data pre-processing of clinical notes

Various techniques were employed to pre-process the clinical notes data. Hexadecimal text and excessive punctuation were removed from the dataset. On average, each patient had approximately 12 notes. To streamline the dataset, notes were sorted chronologically based on their date and time. To overcome the input size limitation of the language models, we employed a summarization technique to extract key information from the notes while retaining as much relevant content as possible. This approach proved more effective than simply truncating the input text to fit the maximum length of the model. The Bart-large variant of Facebook’s BART (Bidirectional and AutoRegressive Transformer) was utilized for summarization [10]. BART-large excels at producing high-quality abstractive summaries due to its bidirectional architecture and autoregressive decoding, making it a useful tool for extracting clear and insightful summaries from longer texts. These summaries were then passed to different models as input.

### 2.3 Development of pre-trained language model-based classifiers

We compared the performance of four different pre-trained language models in the classification of patients appropriate for a nursing-led preoperative phone call assessment. For each language model, we also compared performance with three different data input combinations: (1) diagnosis codes only (D), inputted into the model as their textual representation; (2) clinical text data only (N); and (3) diagnosis codes and clinical text data (D + N). To facilitate model training and evaluation, the dataset was split into a training set (75%) and a test set (25%).

The language models utilized in this study included two general purpose models and two domain-specific models. The general purpose models included Bidirectional Encoder Representations from Transformers (BERT) and A Robustly Optimized BERT Pretraining Approach (RoBERTa) [11, 12]. The two domain-specific language models included versions specifically trained on clinical text and biomedical literature, ClinicalBERT and PubMedBERT, respectively [13, 14]. Further details of each pre-trained language model are provided below.

### 2.4 BERT-base-uncased (BERT)

BERT-Base-Uncased is a language model that makes use of transformer-based architecture [11]. The term “Uncased” denotes that the model was developed using lowercase text and that uppercase and lowercase letters were treated equally during training. The word “Base” in the name of the model alludes to its basic structure. The multiple layers of self-attention mechanisms in BERT-Base-Uncased make it easier to recognize word connections and their context-specific usage. Due to the model’s ability to understand context from both the left and right, this bidirectional approach enables the creation of thorough contextual embeddings. The model picks up contextualized representations of words by anticipating hidden words within a sentence and assessing the coherence between subsequent sentences.

## 2.5 RoBERTa-base (RoBERTa)

This study made use of the RoBERTa-Base model, an expansion of the BERT architecture and pre-training methods [12]. RoBERTa-Base is a developed version of BERT with optimized parameters aimed to improve its performance in a range of NLP tasks. RoBERTa-Base may outperform BERT by making use of larger batch sizes, longer training sequences, and more training data. Its enhanced language representation capabilities may be a result of these improvements.

### 2.6 Bio_ClinicalBERT (ClinicalBERT)

Bio_ClinicalBERT is a specialized transformer-based language model specifically developed for biomedical and clinical text processing [13]. It builds upon the architecture of BioBERT, a pre-trained biomedical language representation model designed for biomedical text mining. Bio_ClinicalBERT undergoes pre-training on a large corpus of EHRs. The Medical Information Mart for Intensive Care-III (MIMIC-III) database, consisting of EHRs from Beth Israel Hospital’s intensive care unit, was then used to train the Bio_ClinicalBERT model [15]. The model can gain domain-specific knowledge and improve its performance in biomedical and clinical text processing tasks due to this sizable corpus of EHR data.

### 2.7 BiomedNLP-PubMedBERT (PubMedBERT)

A transformer-based language model called BiomedNLP-PubMedBERT was created specifically to handle biomedical NLP tasks [14]. It is trained using a sizable corpus of biomedical abstracts obtained from PubMed. For researchers and practitioners working in the biomedical field, this model has shown cutting-edge performance on a variety of biomedical NLP tasks such as knowledge extraction, literature reviews, and the creation of applications that call for the comprehension and processing of biomedical text.

**Fig. 1:**
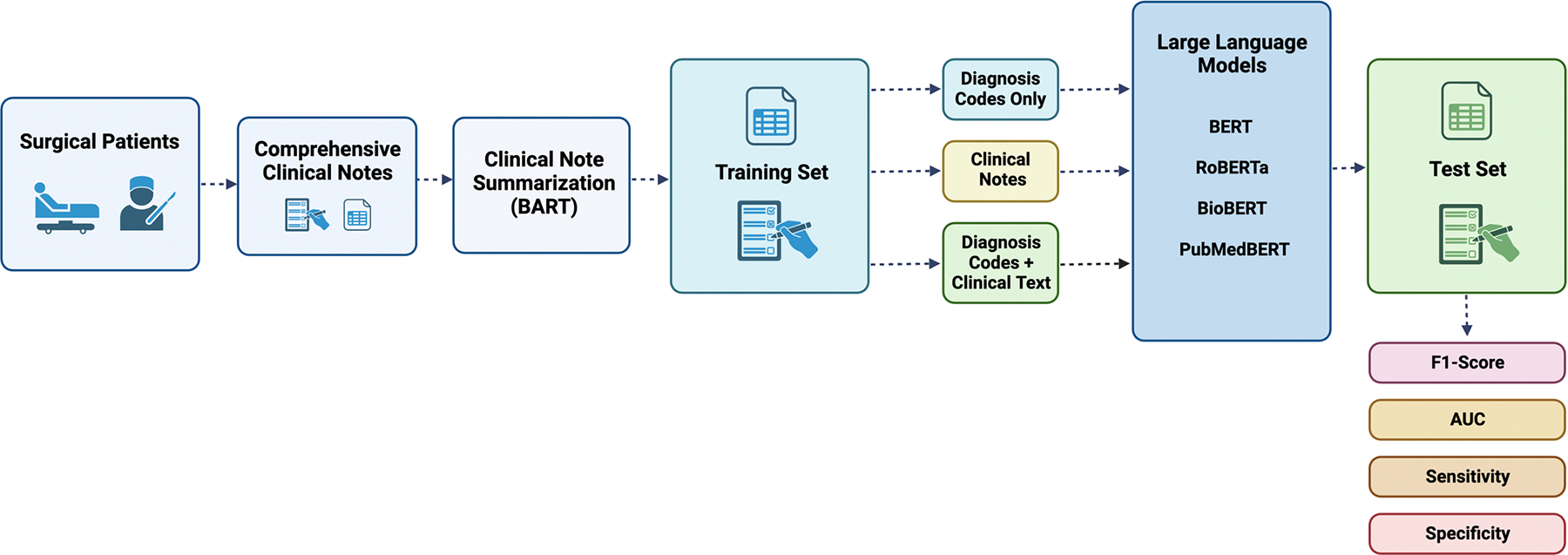
Study design. the study population consisted of surgical patients who were evaluated at the preoperative anesthesia care clinic. clinical notes and diagnosis codes that occurred prior to surgery were extracted for each patient. various language models were used to classify which patients would be appropriate for a nursing preoperative phone screen (rather than in-person/telehealth appointment with an advanced practice provider or anesthesiologist). three combinations of inputs to the models were compared: (1) diagnosis codes only; (2) clinical notes only; versus (3) diagnosis and clinical notes. abbreviations: AUC, area under the receiver operating characteristics curve; BART, bidirectional and autoregressive transformer; BERT, bidirectional encoder representations from transformers; RoBERTa, a robustly optimized BERT pretraining approach

The F1-score, area under the receiver operating characteristic curve (AUC), average precision (AP) based on the precision-recall curve, sensitivity, and specificity were used to assess the performance of each language model. To evaluate each language model’s performance in the given task, these metrics were computed on the designated test set. An illustration of the overall methodology is provided in Fig. 1. Python 3.10.12 was used for all statistical analysis.

#### Data Sharing:

The code for the pre-trained language model-based classifiers is provided in the following link: https://github.com/UCSDGabrielLab/LLMPreoPTriage. The training dataset used was an internal institutional dataset and is not publicly available.

## 3 Results

### 3.1 Study population

The study population consisted of 1,761 unique patients that were scheduled for an evaluation at the preoperative care clinic (Table 1). The total number of clinical documents included in the analysis was 46,922, with an average of 12 notes per patient. The data were split into a training and a test set, which consisted of 1,320 (75%) and 441 (25%) patients, respectively. In the training and test sets, patients that were determined to only require a phone call assessment were 448 (33.9%) and 140 (31.7%), respectively.

### 3.2 Performance of pre-trained language model-based classifiers

**Fig. 2:**
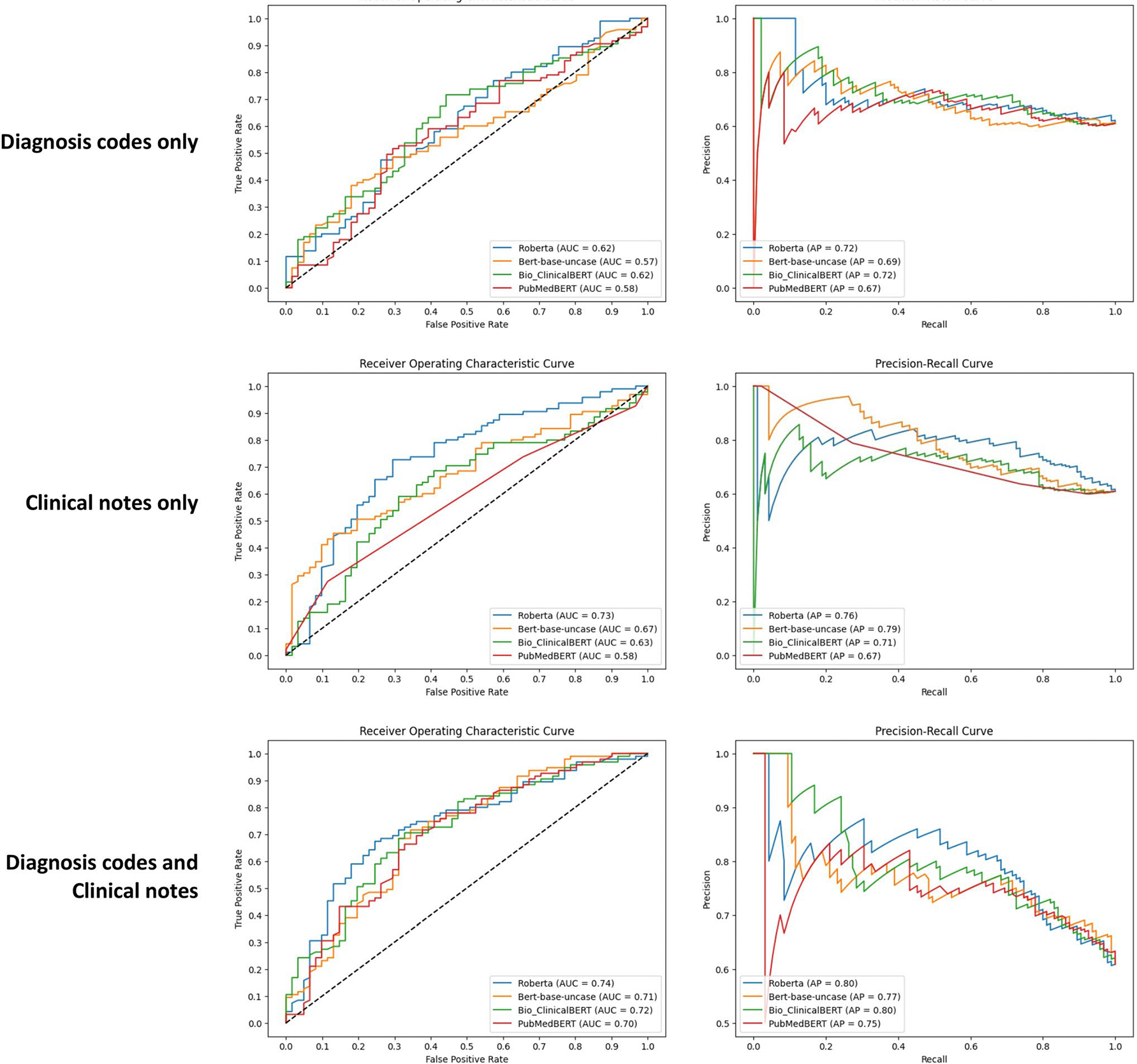
Performance of various language models on the test set in classification of patients using three combinations of inputs: (1) diagnosis codes only; (2) clinical notes only; versus (3) diagnosis and clinical notes. the left column represents area under the receiver operating characteristics curve. the right column represents precision-recall curves. abbreviations: AP, average precision; AUC, area under the receiver operating characteristics curve; BERT, bidirectional encoder representations from transformers; RoBERTa, a robustly optimized BERT pretraining approach

Three combinations of inputs were tested (D, N, and D + N). For each combination, four language models were evaluated (BERT, RoBERTa, ClinicalBERT, and PubMedBERT). Based on the AUC metric, the range between the four language models was highest in the D + N analyses (AUC = 0.70–0.74), lower in the N analyses (AUC = 0.58–0.73) and lowest in the D analyses (AUC = 0.57–0.62). RoBERTa had the highest score compared to the other language models for D (AUC = 0.62), N (AUC = 0.73), and D + N (AUC = 0.74) (Fig. 2).

**Fig. 3:**
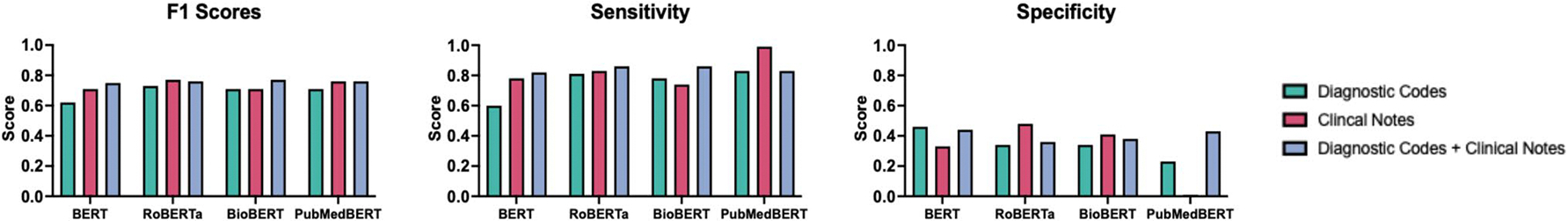
Performance of various language models on the test set in classification of patients using three combinations of inputs: (1) diagnosis codes only; (2) clinical notes only; versus (3) diagnosis and clinical notes. the metrics include F1-score, sensitivity, and specificity

Based on the precision-recall curve, RoBERTa had the highest AP for the D (AP = 0.72) and D + N analyses (AP = 0.80) (Fig. 2). Furthermore, F1-scores, sensitivity, and specificity were reported (Fig. 3). The F1-scores were highest in the D + N analyses (F1 = 0.75–0.77) and lowest in the D analyses (F1 = 0.62–0.71).

## 4 Discussion

Preoperative anesthesia clinics are used to triage the level of assessment a patient needs before surgery. With an increasing demand for preoperative evaluation, language model-based classifiers can be used to assist the decision-making process and improve resource allocation. NLP has been investigated in its use to streamline and automate preoperative evaluation processes [16–19]. Chung, et al. described the use of ClinicalBERT for determining the American Society of Anesthesiologists Physical Status classification score for surgical patients using only free text descriptions from clinical notes [16]. NLP models were also used to accurately detect preoperative cannabis use in perioperative clinical documentation and to predict postoperative mortality [17, 18]. Identification of more than 70 comorbidities as part of surgical patient preanesthestic history using language models has also been reported [19].

Unlike previously reported studies, we described the use of pre-trained language models to triage surgical patients to the type of preoperative evaluation required, suggesting a use for these tools for optimizing workflow and allocating limited resources within the anesthesia preoperative clinic. The results demonstrate an improvement in the classification of a language model-based classifiers for triaging patients when analyzing a combination of clinical notes and diagnosis codes compared to using diagnosis codes alone, suggesting that automated triaging would benefit from methodologies that process both structured and unstructured data. This may be due to the incompleteness or inaccuracy of patients’ problem list, where the diagnosis codes are sourced from [20]. Clinical notes may contain more accurate diagnostic text descriptions of patients’ medical conditions and thus provide valuable information for triaging purposes otherwise not captured from relying solely on structured patient data. Integration of artificial intelligence technology like these language model-based classifiers into clinical workflows may optimize preoperative care [21]. However, widespread adoption of these modalities is limited by several barriers, including more consistent consensus guidelines for the use of artificial intelligence and limitations of infrastructure capabilities [22, 23]. Such algorithms also need further external validation to assess for generalizability and presence of bias [24]. External validation with transfer learning of multimodal models leveraging language modeling is essential for confirming generalizability and stability [25]. Nonetheless, integrating an language model-based classifier for preoperative triaging offers the possibility of reducing nursing workload or full-time equivalent needs in the clinic. Currently, at our institution, triaging of surgical patients is performed by the preoperative anesthesia care clinic nursing team. While some degree of human intervention would likely still be required, as the model discrimination was not perfect, an artificial intelligence-driven tool could potentially reduce the number of nursing staff needed to perform such tasks.

Developing language model-based classifiers for surgical care require several considerations, one of which is choice of the pre-trained language model itself. Here, two general-use language models (BERT, RoBERTa) and two domain-specific language models (ClinicalBERT, PubMedBERT) were assessed. While BERT and RoBERTa offer versatility, their adaptability to the intricacies of medical language may be limited. In contrast, ClinicalBERT, which is specifically fine-tuned on clinical notes, holds promise for tasks involving clinical narratives and diagnosis codes, and can potentially outperform general use models. Similarly, BiomedNLP-PubMedBERT, which is tailored to biomedical literature, may excel in tasks related to research analysis. The choice among these models hinges on which is best suited for the task, with ClinicalBERT and PubMedBERT emerging as strong contenders for applications involving clinical contexts and literature-based inquiries. Ultimately, considerations regarding domain-specific fine-tuning should guide the selection of an appropriate model for robust and accurate medical data analysis. However, this study did not find these domain-specific language models to be superior in performance, which suggests that general use language models, such as RoBERTa, are currently adequate for some clinical tasks when compared to ClinicalBERT.

There are noteworthy limitations to this study related to the aforementioned points about artificial intelligence in medicine. The first point is regarding generalizability of this model. Inter-institution differences in resource limitations and preferences for preoperative anesthesia clinics may result in differences in institution-specific decisions in triaging patients, suggesting the need for future studies validating such models externally to assess performance. This study highlights an important use case: pre-trained language models may be leveraged to “learn” an institution’s preoperative anesthesia care clinic practice patterns and make recommendations based on that institution’s provider preferences. Further advancements of this type of tool can customize the algorithm based on provider preference for preoperative triaging. Additionally, due to the token input limitations of language models, clinical notes had to be pre-processed (i.e. truncated and summarized via BART) for this study. The processing procedure may introduce the loss of important clinical information. As the technology and computational requirements for large language models advance, such limitations may lessen and potentially improve the ability of large language models to capture all relevant clinical information.

In conclusion, we developed language model-based classifiers for triaging preoperative evaluation needs for surgical patients. Inputs that included both structured (diagnosis code descriptions) and unstructured (clinical notes) data were required to optimize performance of these models. As surgical volume grows, so does the need to allocate preoperative resources more efficiently. By leveraging artificial intelligence to aid in this task, clinical workflow may be improved, which could translate to improved surgical outcomes, reduced provider burden, and better resource allocation.

## Figures and Tables

**Table 1:** Basic description of study population

Total patients	1,761
Total notes	46,922
Average notes per patient	12
Age (years), median [quartiles]	60 [45, 70]
ASA score, median [quartiles]	3 [2, 3]
Male sex, n (%)	817 (46.4)
Body mass index (kg/m2), median [quartiles]	26.6 [23.1, 30.8]

ASA, American Society of Anesthesiologists

## Data Availability

The code for the pre-trained language model-based classifiers is provided in the following link: https://github.com/UCSDGabrielLab/LLMPreoPTriage. The training dataset used was an internal institutional dataset and is not publicly available but is available with appropriate data use agreements in place.

## Abbreviations

AP: average precision
AUC: area under the receiver operating characteristics curve
BART: Bidirectional and autoregressive transformer
BERT: Bidirectional encoder representations from transformers
D: diagnosis codes only cohort
D + N: diagnosis codes and clinical text data cohort
EHR: electronic health record
N: clinical text data only cohort
NLP: natural language processing
RoBERTa: A robustly optimized BERT pretraining approach
